# Effect of pregnancy on female gait characteristics: a pilot study based on portable gait analyzer and induced acceleration analysis

**DOI:** 10.3389/fphys.2023.1034132

**Published:** 2023-05-16

**Authors:** Xin Li, Zhenghui Lu, Xuanzhen Cen, Yizheng Zhou, Rongrong Xuan, Dong Sun, Yaodong Gu

**Affiliations:** ^1^ Faculty of Sports Science, Ningbo University, Ningbo, China; ^2^ Faculty of Engineering, University of Szeged, Szeged, Hungary; ^3^ The Affiliated Hospital of Medical School, Ningbo University, Ningbo, China; ^4^ Research Academy of Medicine Combining Sports, Ningbo No 2 Hospital, Ningbo, China

**Keywords:** pregnant women, induced acceleration, OpenSim, IDEEA, gait analysis

## Abstract

**Introduction:** The changes in physical shape and center of mass during pregnancy may increase the risk of falls. However, there were few studies on the effects of maternal muscles on gait characteristics and no studies have attempted to investigate changes in induced acceleration during pregnancy. Further research in this area may help to reveal the causes of gait changes in women during pregnancy and provide ideas for the design of footwear and clothing for pregnant women. The purpose of this study is to compare gait characteristics and induced accelerations between non-pregnant and pregnant women using OpenSim musculoskeletal modeling techniques, and to analyze their impact on pregnancy gait.

**Methods:** Forty healthy participants participated in this study, including 20 healthy non-pregnant and 20 pregnant women (32.25 ± 5.36 weeks). The portable gait analyzer was used to collect participants’ conventional gait parameters. The adjusted OpenSim personalized musculoskeletal model analyzed the participants’ kinematics, kinetics, and induced acceleration. Independent sample T-test and one-dimensional parameter statistical mapping analysis were used to compare the differences in gait characteristics between pregnant and non-pregnant women.

**Results:** Compared to the control group, pregnancy had a 0.34 m reduction in mean walking speed (*p* < 0.01), a decrease in mean stride length of 0.19 m (*p* < 0.01), a decrease in mean stride frequency of 19.06 step/min (*p* < 0.01), a decrease in mean thigh acceleration of 0.14 m/s^2^ (*p* < 0.01), a decrease in mean swing work of 0.23 g (*p* < 0.01), and a decrease in mean leg falling strength of 0.84 g (*p* < 0.01). Induced acceleration analysis showed that pregnancy muscle-induced acceleration decreased in late pregnancy (*p* < 0.01), and the contribution of the gastrocnemius muscle to the hip and joint increased (*p* < 0.01).

**Discussion:** Compared with non-pregnant women, the gait characteristics, movement amplitude, and joint moment of pregnant women changed significantly. This study observed for the first time that the pregnant women relied more on gluteus than quadriceps to extend their knee joints during walking compared with the control group. This change may be due to an adaptive change in body shape and mass during pregnancy.

## 1 Introduction

Walking is the most common way of exercise and an essential part of daily life for pregnant women (Bassett and Tudor-Locke, 2004). During pregnancy, the physiological and morphological changes in women are accompanied by changes in mass distribution to accommodate the growing size and mass of the fetus (Aguiar et al., 2015; Ogamba et al., 2016; Ren et al., 2020). These changes in body shape and proportions have been shown to be closely associated with many disease incidence and mortality factors (Bogin and Varela-Silva, 2010). For example, changes in height and weight may affect the occurrence of diseases such as osteoporosis, cardiovascular disease, diabetes, cancer, and others. This is crucial for current research as it can help us better understand the relationship between bodily changes and health, thereby improving our ability to prevent and treat these diseases. For pregnant women, these changes are also may cause cardiovascular and hormonal changes (Collings et al., 2020; Gao et al., 2020; Lu H. et al., 2022), which may lead to joint laxity as well as fluid retention and compression of soft tissues (Haddox et al., 2020). These changes may cause musculoskeletal pain (Kesikburun et al., 2018) and lead to changes in gait and increase the risk of falls (Haddox et al., 2020; Song et al., 2020). According to the current international research about low back and pelvic pain during pregnancy, more than half of pregnant women reported hip, knee, or foot pain (Wang et al., 2004; Wu et al., 2004; Ng et al., 2017).

Gait parameters provide a good indication of physical coordination and other abilities (Kalron et al., 2020). Compared with non-pregnant women, pregnant women showed special gait characteristics (Mei et al., 2018). At present, Błaszczyk et al. (2016) have analyzed the spatiotemporal gait characteristics of pregnant women. They found a 0.14 m/s reduction in walking speed, a 0.09 cm reduction in stride length, and a 20 m increase in double support time in late pregnancy compared to the 6 postpartum period.

However, the factors causing this change in gait characteristics are still not very clear. Błaszczyk et al. (2016) believe that this change is mainly caused by changes in body weight during pregnancy and fear and anxiety of losing balance. In a study involving the kinetics of lower limbs, Bagwell et al. (2020) found that pregnant women had greater ankle moments and less activation of the gluteus maximus during walking. Therefore, this change in gait characteristics may be related to changes in musculoskeletal adaptation during pregnancy, and it is essential to understand the biomechanical factors that alter the gait characteristics of pregnant women during exercise.

Human exercise involves complex interactions between muscles and bones (Gu et al., 2013; Rajagopal et al., 2016; Zhang et al., 2023). Muscles are force-producing entities and, as such, kinetic data and muscle forces are often considered indicators of kinetic muscle function (Kimmel and Schwartz, 2006), there is evidence that forces affect activity across joints and segments (Arnold et al., 2005; Kimmel and Schwartz, 2006). Besides, anatomy alone may not be sufficient to describe the effects of muscles on body posture and joint movement (Souza et al., 2022). For example, in walking, the gluteus maximus muscle may have an effect similar to that of the quadriceps femoris, affecting the knee joint’s acceleration (Neptune et al., 2004; Arnold et al., 2005). It may even have the opposite effect on anatomy (Neptune et al., 2004). Induced acceleration analysis (IAA) is a method that can be calculated to analyze the acceleration caused by force on an object or system (Schwartz and Lakin, 2003), which can quantify the effect of muscle contraction on body joints during exercise (Kimmel and Schwartz, 2006). In the study of Souza et al. (2022), the author described the joint-induced acceleration caused by the main lower limb muscles during static standing and walking. Souza et al. (2022) found that specific bi-articular muscles such as the soleus and gastrocnemius at the ankle joint and the iliopsoas at the knee joint exhibited the opposite of their anatomical function, and they hypothesized that this phenomenon might be a compensatory phenomenon to maintain movement stability. Changes in gait may occur if there is inadequate compensation, muscle weakness or dysfunction (Correa et al., 2012). This phenomenon may be related to changes in the gait pattern of women during pregnancy. However, due to the physiological changes caused by pregnancy, the results of the analysis of the general population do not apply to pregnant women. Therefore, it is essential to independently analyze pregnant women and evaluate their relationship with gait characteristics.

As mentioned, gait tests were usually conducted in the gait laboratory (Błaszczyk et al., 2016; Zhang et al., 2016; Mei et al., 2018; Bagwell et al., 2020; Haddox et al., 2020; Chaitanya and Sekhar, 2021; Patra et al., 2022). However, the laboratory environment may affect the gait characteristics of participants, and participants’ gait parameters may be affected by psychological discomfort with the laboratory environment. And a single or small amount of gait data collected in the laboratory environment may not fully reflect the real situation (Sun et al., 2018; Akhavanhezaveh and Abbasi-Kesbi, 2021), and there are some limitations in the experimental site (Zhang et al., 2021). Therefore, in this study, we use a portable gait analyzer (IDEEA, MiniSun, Fresno, CA, United States) to collect the gait data of participants.

The portable gait analyzer is a portable device that uses accelerometers and gyroscopes to measure the type, duration, intensity and frequency of physical activity. It can be used to objectively and quantitatively monitor the participants’ walking movements at any time, regardless of the location, allowing for a more realistic recording of walking in life. Sun et al. (2018) demonstrated the reliability of the IDEEA system for gait analysis. The study revealed that the measurements of gait cycle, cadence, step length, velocity, and number of steps obtained using a GoPro (sampling frequency of 60 Hz) camera were not statistically different (*p* > 0.05) from those obtained with the IDEEA system. Combined with the manufacturer’s claims (Saremi et al., 2006) that the device can be effective in assessing gait parameters.

In the process of pregnancy, changes in physiological morphology will affect the characteristics of the female gait. Because of these altered physiological patterns, pregnant women may experience some degree of compromised stability during walking (Aguiar et al., 2015; Ogamba et al., 2016; Haddox et al., 2020), and may experience musculoskeletal pain (Schwartz and Lakin, 2003; Neptune et al., 2004; Wang et al., 2004; Wu et al., 2004; Arnold et al., 2005; Kimmel and Schwartz, 2006; Błaszczyk et al., 2016; Rajagopal et al., 2016; Ng et al., 2017; Mei et al., 2018; Bagwell et al., 2020; Souza et al., 2022). However, the causes of these symptoms are still not fully understood, and for this reason, Branco et al. (2014) also suggest that research could focus more on changes in kinetics and muscle engagement in women during pregnancy, to further understand how these changes affect gait parameters in pregnant women. Therefore, this study uses OpenSim musculoskeletal modeling technology to compare the gait parameters and joint-induced acceleration between non-pregnant women and pregnant women and explore the relationship between induced acceleration and gait parameters. Based on the results of previous studies, we hypothesize that: 1) The gait parameters of pregnant women differ from those of non-pregnant women and account for a greater proportion of reduced gait speed and double support periods. 2) Pregnant women have reduced joint mobility and reduced peak joint moments. 3) The contribution of lower limb muscle groups to joint motion (i.e., induced acceleration) may differ in pregnant women.

## 2 Materials and methods

### 2.1 Participants

Table 1 presents the basic characteristics of the participants, referring to the study by Bagwell et al. (2020), Forty healthy participants participated in this study, including 20 healthy non-pregnant women (age:27.00 ± 4.58) and 20 pregnant (age: 29.00 ± 3.94 and gestational: 32.25 ± 5.36 weeks). The participants were healthy and had not undergone lower limb or back surgery. During pregnancy, as the gestational period progresses, pregnancy pelvis widens and weight increases. Therefore, to increase stability during walking, pregnant women should shorten their stride and reduce their walking speed, resulting in a unique gait pattern.

**TABLE 1:** Basic characteristics of the participants.

Group	N	Gestational age (weeks)	Waist Circumference (m)	Age (yr)	Weight (kg)	Height (m)	BMI (kg/m^2^)
CON	20	/	/	27.00 ± 4.58	56.10 ± 4.39	1.65 ± 0.05	20.56 ± 1.35
Pregnant woman	20	32.25 ± 5.36	0.98 ± 0.07	29.00 ± 3.94	67.00 ± 7.51	1.66 ± 0.05	25.30 ± 3.68

CON, Control group. The BMI data in the table were measured when the participants underwent laboratory testing.

The participants’ height, weight, and waist circumference were measured before the test, and the pregnant women were asked about their pre-pregnancy height and weight, with all participants having a pre-pregnancy body mass index (BMI) of less than 26 kg/m^2^. All pregnant participants included in the study were first pregnant. In addition, 20 non-pregnant women aged 23–37 years who met the requirements were recruited as a control group. All pregnant participants were recruited by the researchers through questionnaires and interviews at the School of Medicine Affiliated Hospital of Ningbo University. Non-pregnant participants were also recruited by other researchers through questionnaires and interviews in the local area. Again, non-pregnant women were excluded if they had undergone lower limb or back surgery, or were unable to take the gait test. All of them filled out the informed consent form and were informed of the complete experimental procedure and the purpose of the study. The study was approved by the Ethics Committee of Ningbo University (RAGH202201154396.6).

### 2.2 Experimental design

**FIGURE 1:**
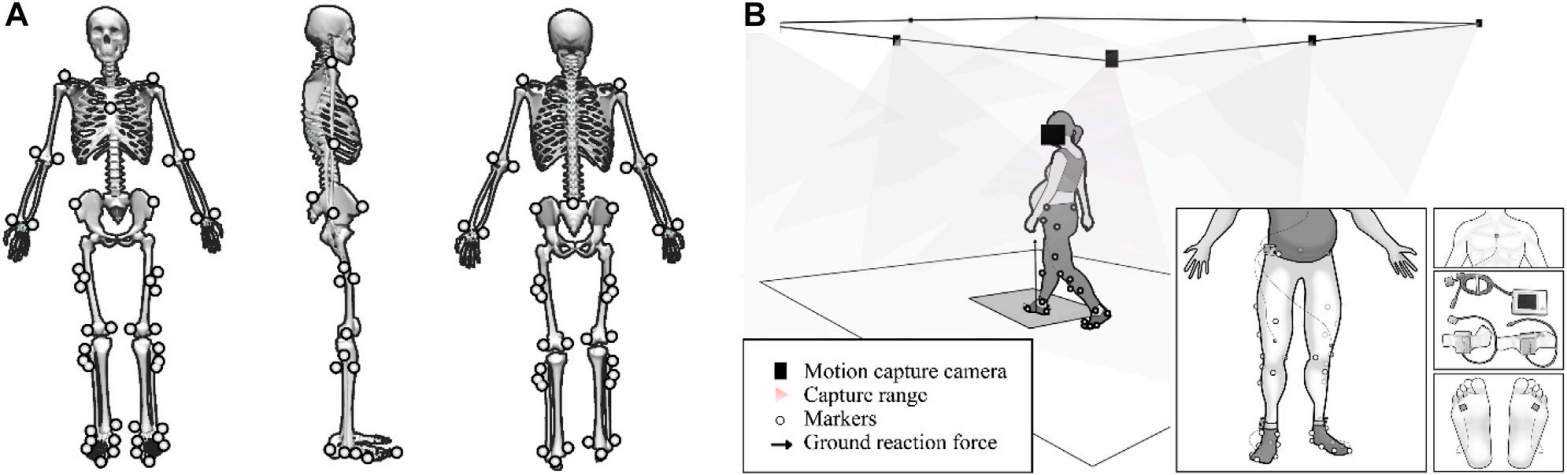
**(A)**, mark paste position. **(B)**, schematic of the test site and the portable gait analyzer.

Gait parameters were measured using the IDEEA 3 (IDEEA, MiniSun, Fresno, CA, United States), which has a sampling frequency of 32 Hz. Participants were asked to walk 30 m at a self-selected comfortable pace, while gait parameters were collected by main recorders. IDEEA software was used to analysis the middle 20 steps’ gait parameters. Specifically, the device was used to collect data on walking speed, step frequency, stride length, thigh acceleration, swing work, leg falling strength, and single/double support time. As shown in Figure 1B, IDEEA 3 consisted of a main recorder on the lateral waist and two subrecorders on the side of the ankle. The main recorder was connected to three limb sensors that were attached to the sternum and mid-thigh, and two sub-recorders were each connected to a limb sensor that was attached to the each side of fourth metatarsophalangeal joint. Each sensor can measure the angular changes of body segments and the motion (acceleration) in three orthogonal directions independently. These 5 sensors eventually send data to the recorder to record movement data during exercise. IDEEA is easy to wear and operate, is field-independent and causes little disruption to participants’ walking.

As shown in Figure 1B, the Vicon3D motion capture system (Vicon Metrics Ltd, Oxford, uk) consists of eight 1.3 MP pixel (1280*1024) infrared cameras and an AMTI 3D force plate (AMTI, Watertown, Massachusetts, United States). The eight cameras will record the participants’ body movements during walking at 200 Hz, while the force plate will record the ground reaction force data at 1000 Hz. Data from cameras and force plates of the participants during walking were put into OpenSim to calculate joint-induced accelerations.

Before the test, participants wore a portable gait tester and affixed 46 markers, then walked freely around an open and flat area to familiarise themselves with the environment and equipment. The markers are pasted in the positions shown in Figure 1A. The test site has an area of 500 m^2^ and is covered with a hard, solid wooden floor. To avoid unnecessary errors, the participants were uniformly provided with the same type of sports shoes and socks. During the test participants were asked to walk at a comfortable pace on the same course and gait data was collected using IDEEA. Participants then passed through the force platform at their own pace and without instruction. Kinematic and kinetic data were collected from the subjects and five successes were collected for each subject. Successful data was defined as the participant completing the gait test in a position that was considered natural and comfortable for them, without missing or obscuring the reflective marker points affixed to their body, and without stepping off the force platform while passing over it with their foot in the center of the force platform.

### 2.3 Modelling and simulation

As pregnancy can lead to changes in body mass (Bodnar et al., 2015), and the distribution of segmental mass is very different from that of a common mannequin. So this study used a specific modified OpenSim musculoskeletal model for pregnant women (Haddox et al., 2020), which adjusted the mass and centroid position of different parts of women during different pregnancies.

The trajectory of the marked point and the ground reaction force data are converted by the self-made Matlab program. First, running OpenSim’s scaling tool and then manually adjusting the weights and positions of the markers (as in Figure 1A) on the body segments in the OpenSim gait model based on the initial results to match the positions of the markers in the actual experiments to reduce errors.

The model is scaled to meet anthropometry characteristics so that the root mean square error between the marked experimental point and the virtual marked point is less than 0.02 m, and the maximum error is less than 0.04 m (Lu et al., 2022b; Lu et al., 2022c; Gao et al., 2022). The inverse kinematics algorithm calculates the joint angle, and the residual reduction algorithm reduces the kinetics inconsistency between kinematics and ground reaction force. In this case, inverse kinematics solves the weighted least squares problem to minimize the marker positions in each frame of the simulation and experiment (Lu and O’connor, 1999; Pizzolato et al., 2017). The joint moment is calculated using inverse dynamics. Using computed muscle control (CMC) to estimate muscle activation and muscle-tendon force during exercise (Delp et al., 2007). The CMC algorithm accurately tracks the joint angles of the participant during the test with only small deviations from kinematic and ground reaction forces and can generate muscle-driven simulations quickly and accurately (Thelen and Anderson, 2006).

### 2.4 Induced acceleration analysis (IAA)

Use the analysis tool to run IAA to calculate the IA (rad/s^2^/N/kg) and IA (rad/s^2^) per unit of force generated by the muscle. Induced acceleration is calculated as the acceleration generated by the muscle force for each isolated muscle group. It is worth noting that since a joint can be subjected to multiple forces at the same time, and the forces can superimpose or cancel each other out (Chen, 2006; Souza et al., 2022), so the induced acceleration is not a joint acceleration in the actual sense. The actual joint acceleration can be expressed as the sum of all muscle and non-muscle induced acceleration (Zajac and Gordon, 1989; Souza et al., 2022). In the analysis tool, OpenSim provides several constraint types that are supported with an Induced Acceleration Analysis, including Point, Weld, and Rolling on Surface constraints. A Point constraint forces two points on separate bodies to remain coincident, but allows free relative rotation about that point; a Weld is similar, but also constrains the orientation of the two bodies to remain fixed to one another. A Rolling On Surface describes a constraint on a rolling body, that is, in contact with a plane defined on another body. In this study, the foot–floor interaction was modeled with a rolling without slipping constraint (Hamner et al., 2010; Hamner and Delp, 2013a; Hamner et al., 2013b).

The sum of the acceleration of the centre of mass due to muscle force, gravity and velocity effects was then compared to the experimentally measured acceleration of the centre of mass to verify the accuracy of the analysis (Hamner et al., 2010; Hamner and Delp, 2013a).

### 2.5 Outcomes measurement

The gait parameters evaluated in the study are as follows (Maffiuletti et al., 2008; Lu et al., 2021): 1) Walking speed (m/s): the distance of walking per unit of time. 2) Stride length (m): the distance between two touchdowns on one heel. 3) Step frequency: steps per minute. 4) Thigh acceleration (g/1g = 9.8 m/s^2^): at the beginning of the swing phase, the average acceleration of the thigh segment is defined as the thigh acceleration, which indicates the strength of the thigh during thigh pumping. 5) Swing work (g/1g = 9.8 m/s^2^): the average acceleration of the foot during the swing phase and the work of the swing represents the strength of the swing process. 6) Leg falling strength (g/1g = 9.8 m/s^2^): the average acceleration of the foot during the acceleration descent at the end of the swing phase, indicating the degree of force at the moment of landing. 7) Single support time/double support time (%): the ratio of the time spent using one-foot support to the time spent using biped support in a gait cycle, reflects the stability of the participants when walking. The average curve was used to describe the joint-induced acceleration caused by muscle-tendon force during the walking stance phase, and the average, maximum and minimum values were calculated. The test results are expressed by average ± standard deviation (mean ± SD).

OpenSim was used to calculate the peak joint angle, range of motion, peak joint moment, muscle force, and induced acceleration of participants during the walking support period**.** The main muscle groups calculated include gluteus, iliopsoas, hamstrings, quadriceps, soleus, gastrocnemius, and tibialis, which may lead to changes in gait characteristics (Souza et al., 2022). Because the opposite moments cancel each other out (Chen, 2006), the joint acceleration calculated by IAA is not the actual acceleration. The actual joint acceleration can be calculated as the sum of all induced acceleration caused by muscle and non-muscle. This study used mean and standard deviation curves to represent muscle-tendon force, induced acceleration, and joint angular acceleration per unit force expressed as a percentage of the stance phase. As hip flexion, knee extension, and ankle dorsiflexion are by default positive in this model of the OpenSim software, hip flexion, knee extension, and ankle dorsiflexion are also by default positive in this study. For example, the greater the degree of hip flexion, the greater the angle value, with the maximum value being the angle of flexion at maximum flexion. Because of the fluctuation of the value in each acceleration curve, the average, minimum and maximum values of the walking stance phase are calculated.

### 2.6 Statistical analysis

After the data collection, the data is saved in the primary recorder, downloaded to the computer, and further analyzed using IDEEA Version 3.01 (IDEEA3; MiniSun). SPSS 21.0 statistical software package (SPSS, Chicago, Illinois, United States) was used for statistical analysis. Normality hypotheses was verified using Shapiro-Wilk’s tests, and the differences in average, maximum, minimum, and gait data of induced acceleration between non-pregnant women and pregnant women were analyzed by an independent sample T-test. Matlab (MATLAB R2017a, MASS, Natick, MA, United States) was used to run the open-source one-dimensional parameter statistical mapping program (SPM1D) to analyze the continuous data. The significance level was set to 0.01.

## 3 Results

**FIGURE 2:**
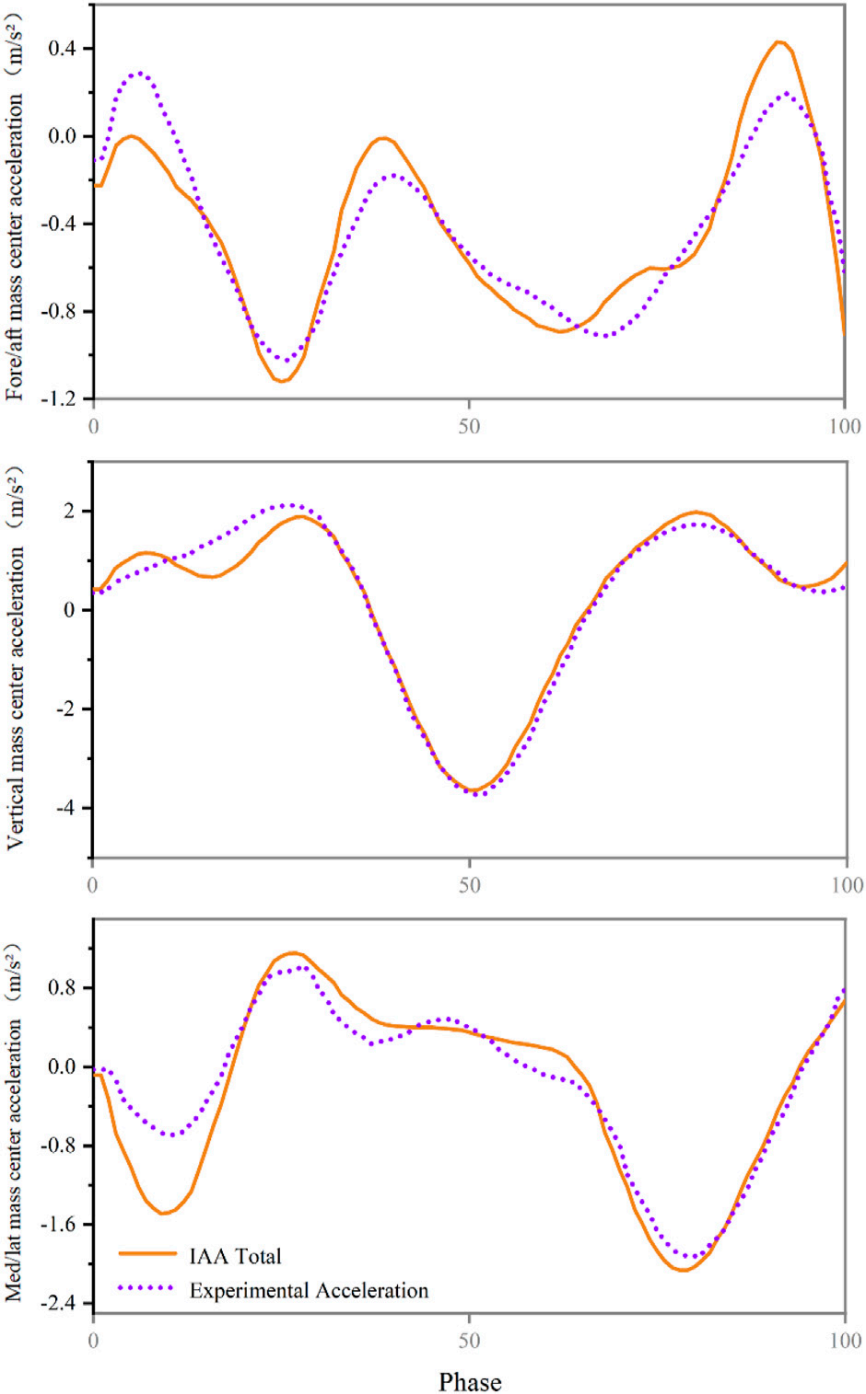
Compare the acceleration of the centre of mass calculated from experimental measurements (purple) with the acceleration of the centre of mass calculated by induced acceleration analysis (orange).

We verified that the sum of all accelerations due to muscle, gravity and velocity effects equals the total acceleration of the centre of mass (Figure 2).

### 3.1 Gait parameters

The comparison of gait parameters between non-pregnant women and pregnant women is shown in Table 2. The results showed that compared to no-pregnant, pregnancy had a mean decrease in walking speed of 0.34 m/s (*p* < 0.01), a mean decrease in stride length of 0.19 m (*p* < 0.01), a mean decrease in stride frequency of 19.06 step/min (*p* < 0.01), a mean decrease in thigh acceleration of 0.14 m/s^2^ (*p* < 0.01), a mean decrease in an swing work of 0.23 g (*p* < 0.01), and a mean decrease in leg falling strength of 0.84 g (*p* < 0.01).

**TABLE 2:** Gait parameters of non-pregnant and pregnant women.

Index	Pregnant woman	CON	t	p
Walking speed (m/s)	0.83 ± 0.16*	1.17 ± 0.07	−8.71	*p* < 0.01
Stride length (m)	1.05 ± 0.07*	1.24 ± 0.04	−10.54	*p* < 0.01
Step frequency (Step/minute)	93.41 ± 11.89*	112.47 ± 3.79	−6.83	*p* < 0.01
Thigh acceleration (g/1g = 9.8 ${m/s}^{2}$ )	1.02 ± 0.05*	1.16 ± 0.10	−5.60	*p* < 0.01
Swing work (g/1g = 9.8 ${m/s}^{2}$ )	0.48 ± 0.25*	0.71 ± 0.18	−3.92	*p* < 0.01
Leg falling strength (g/1g = 9.8 $\left.{m/s}^{2}\right)$	1.16 ± 0.54*	2.00 ± 0.18	−6.60	*p* < 0.01
Single support time/double support time	3.09 ± 0.18	3.45 ± 0.50	−2.32	*p* = 0.03

CON, Control group, i.e., non-pregnant women.

*Significant differences in this index between non-pregnant and pregnant women.

In addition, even if there was no statistically significant difference, the single support time/double support time of pregnant women was less than those of non-pregnant women.

### 3.2 Joint angle and joint moment

In this study, the degree of muscle activation calculated by OpenSim simulation is similar to that of previous studies (Lin et al., 2012; Trinler et al., 2019), indicating that the data of the OpenSim model in this study are reliable.

The participants’ peak joint angle, joint moment, and joint range of motion during the stance phase of walking are shown in Table 3. The results showed that the range of motion of the hip joint, knee joint, and ankle joint in the pregnant women was lower than that in the control group. Pregnant people have approximately 11° decreased hip ROM compared to control (*p* < 0.01). In addition, during the gait stance phase, the peak flexion and extension moment of the hip joint, knee joint, and ankle joint of pregnant women were significantly lower than those in the control group (*p* < 0.05).

**TABLE 3:** Peak joint angle, joint moment, and joint range of motion.

Index		Pregnant woman	CON	t	p
Hip	Max angle (°)	46.27 ± 1.74	39.33 ± 10.41	2.94	*p* = 0.01
	Min angle (°)	11.12 ± 2.94*	−6.61 ± 6.33	11.36	*p* < 0.01
	ROM (°)	35.14 ± 3.21*	45.94 ± 9.24	−4.94	*p* < 0.01
	Extension moment (Nm/kg)	0.48 ± 0.08*	1.18 ± 0.60	−5.17	*p* < 0.01
	Flexion moment (Nm/kg)	0.28 ± 0.10*	0.94 ± 0.16	−15.64	*p* < 0.01
Knee	Max angle (°)	−19.76 ± 4.77*	−6.42 ± 3.51	−10.07	*p* < 0.01
	Min angle (°)	−69.56 ± 10.50*	−59.11 ± 10.26	−3.18	*p* < 0.01
	ROM (°)	49.77 ± 11.64	52.69 ± 12.87	−0.75	*p* = 0.46
	Extension moment (Nm/kg)	0.48 ± 0.16*	1.00 ± 0.22	−8.55	*p* < 0.01
	Flexion moment (Nm/kg)	0.17 ± 0.08*	0.58 ± 0.28	−6.30	*p* < 0.01
Ankle	Max angle (°)	28.46 ± 3.63*	13.80 ± 7.14	8.19	*p* < 0.01
	Min angle (°)	−0.93 ± 10.74*	−23.52 ± 10.05	6.87	*p* < 0.01
	ROM (°)	29.39 ± 11.07	37.32 ± 10.20	−2.36	*p* = 0.02
	Plantarflexion moment (Nm/kg)	1.20 ± 0.06*	1.27 ± 0.08	−3.13	*p* < 0.01
	Dorsiflexion moment (Nm/kg)	0.12 ± 0.04*	0.45 ± 0.12	−11.67	*p* < 0.01

CON, Control group, i.e., non-pregnant women.

*Significant differences in this index between non-pregnant and pregnant women.

**FIGURE 3:**
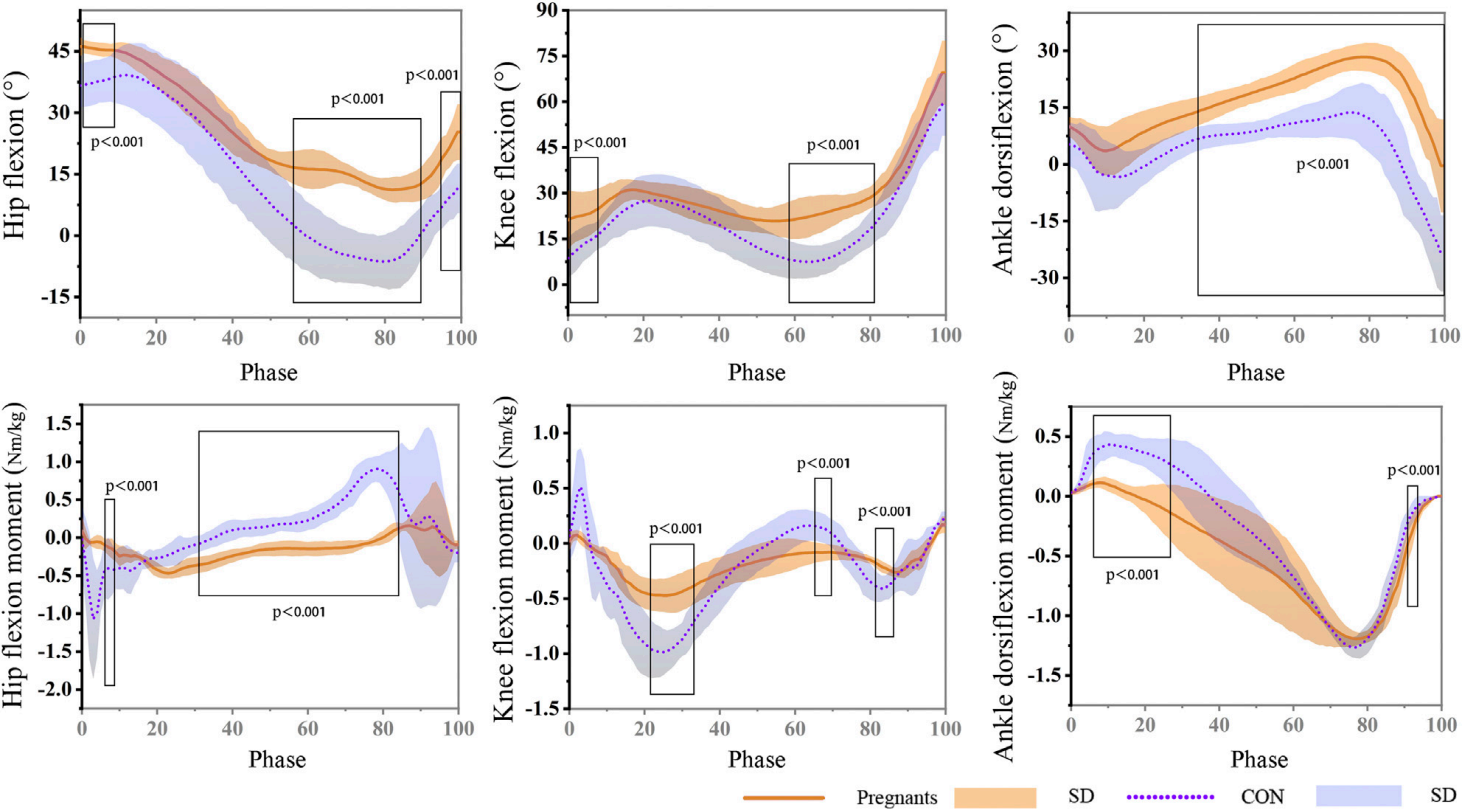
During the gait stance phase, the hip, knee, and ankle angle and moment of pregnant (orange) and non-pregnant (purple).

The data of the participants’ joint angle and moment during the gait stance phase are shown in Figure 3. The results showed that the angle of hip flexion in the pregnant women was significantly larger than that in the control group during the gait stance phase (0%–9%) and pedal off stage (56%–90%; 95%–100%) (*p* < 0.001). It is worth noting that the hip joint of pregnant women still showed flexion in the late support phase, while the control group was in the state of extension. The knee flexion angle of the participants in the pregnant women was significantly larger than that in the control group, and it was statistically significant at the stages of 0%–8% and 58%–80% of the stance phase (*p* < 0.001). During the support stage, the hip joint angle of the participants was flexion at first and then extended. The ankle flexion of pregnant women in the second half of the support phase (34%–100%) was significantly higher than that of the control group (*p* < 0.001), and the extension angle is significantly smaller (*p* < 0.001). In addition, in the early stage of landing during the stance phase (3%–5%), the hip extension moment of pregnant women was significantly lower than that of the control group (*p* < 0.001), and at the stage of 28%–82% of the stance phase, the hip joint extension moment was significantly larger than that of the control group (*p* < 0.001). In the stages of 19%–30%, 62%–67%, and 78%–83% of the stance phase, pregnant women’s knee joint flexion and extension moment were significantly lower than the control group (*p* < 0.001). During the stance phase, the ankle dorsiflexion moment of pregnant women was significantly higher than the control group (4%–26%) (*p* < 0.001). At the 90%–92% stage, the ankle plantarflexion moment was significantly larger than the control group (*p* < 0.001).

### 3.3 Induced acceleration analysis

**FIGURE 4:**
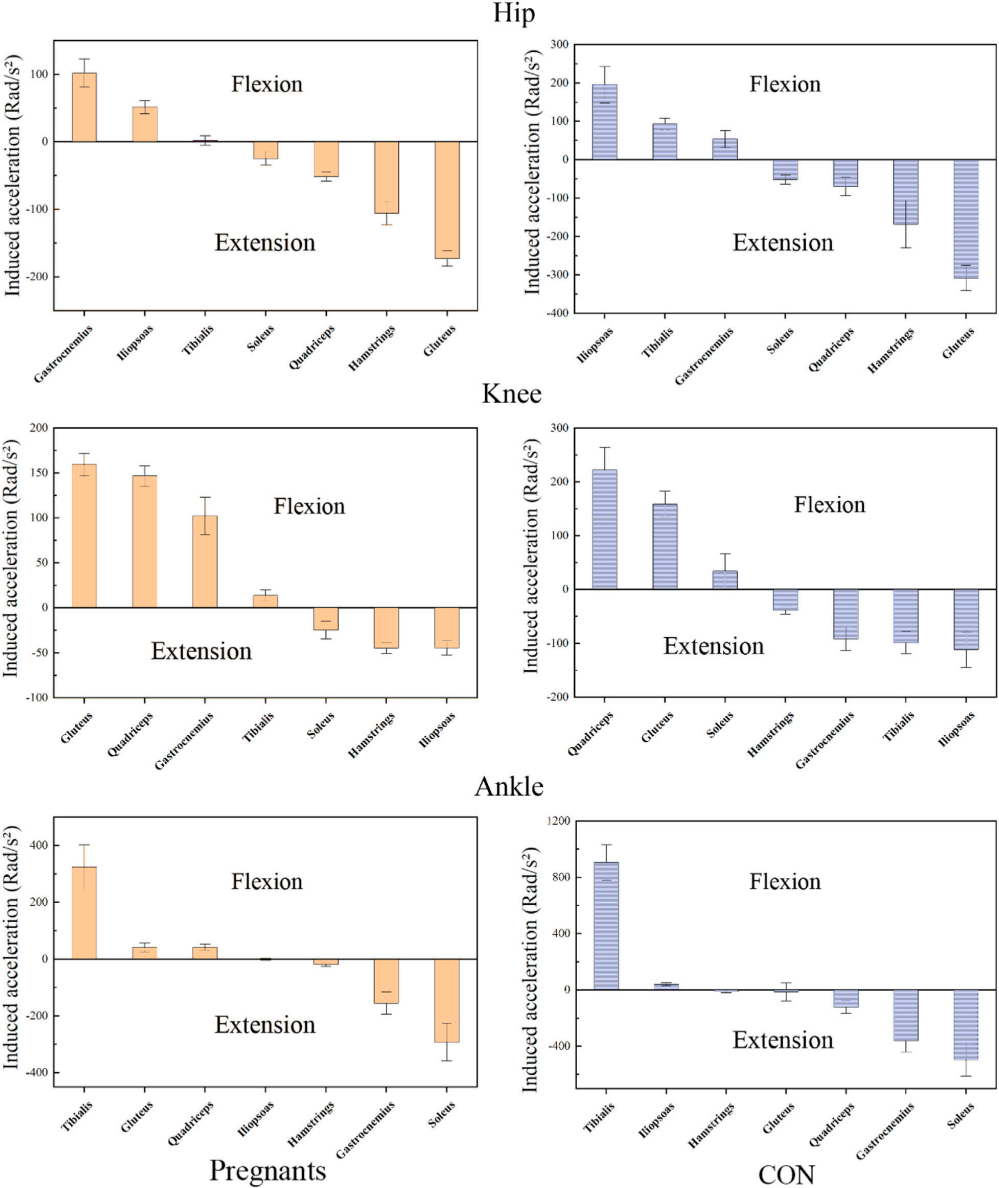
The average induced acceleration of pregnant (orange) and no-pregnant (purple) in the gait stance phase.

**FIGURE 5:**
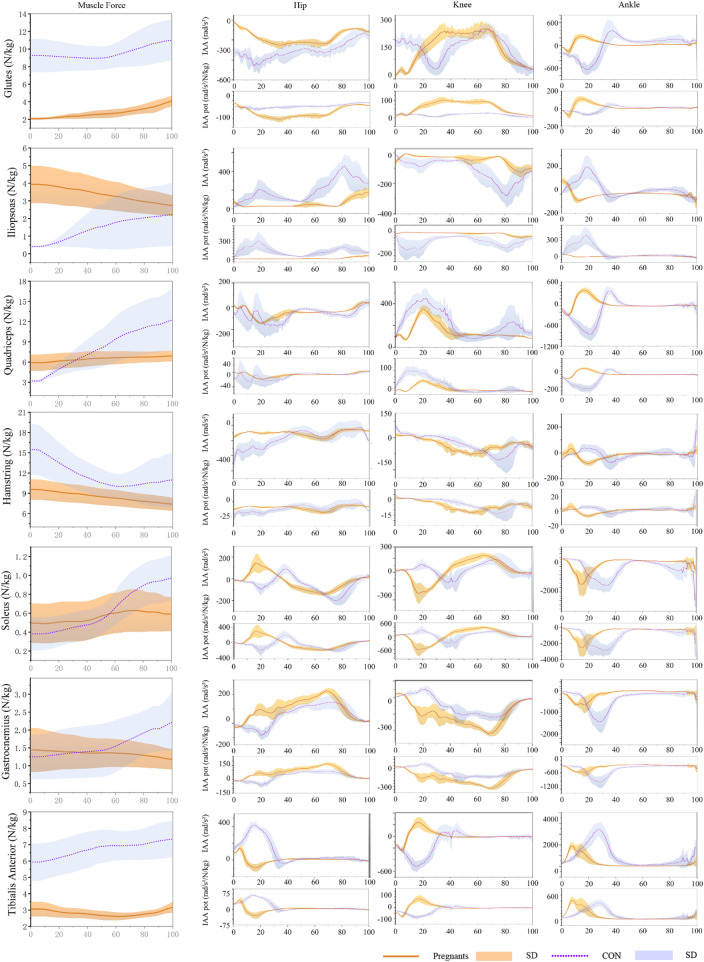
During the gait stance phase, standardized muscle-tendon force, induced acceleration per unit force, and induced acceleration of pregnant (orange) and non-pregnant (purple) were expressed as mean and standard deviation.

The induced acceleration of the participants during the stance phase of walking is shown in Figure 4; Table 4, and the data of muscle force, induced acceleration, and induced acceleration per unit force are shown in Figure 5. About the direction of gait support, gluteus, quadriceps, hamstring, and soleus muscles were the primary hip extensor muscles in the pregnancy group and control group, that is, they accelerated hip extension; gastrocnemius, tibialis anterior muscle, and iliopsoas muscle were the main hip flexion groups, accelerating hip flexion. Gluteus and quadriceps femoris are the main knee extensor groups that accelerate knee extension; while hamstring and iliopsoas muscles are the main knee flexion groups that accelerate knee flexion. However, gastrocnemius and tibialis anterior muscle showed different effects in the pregnancy and control groups.

**TABLE 4:** Mean induced accelerations at the joints during the stance phase.

Index			Pregnant	Con	t	p
Glutes	Hip (rad/s^2^)	Max	−19.47 ± 4.58*	−122.66 ± 35.22	12.99	*p* < 0.01
		Min	−280.92 ± 42.01*	−530.11 ± 55.04	16.09	*p* < 0.01
		Average	−172.59 ± 11.36*	−308.03 ± 33.20	17.26	*p* < 0.01
	Knee (rad/s^2^)	Max	287.46 ± 42.67	284.03 ± 28.59	0.30	*p* = 0.77
		Min	13.31 ± 3.00	15.47 ± 21.96	−0.44	*p* = 0.67
		Average	159.45 ± 12.51	158.55 ± 24.50	0.15	*p* = 0.88
	Ankle (rad/s^2^)	Max	251.67 ± 64.80*	505.54 ± 198.43	−5.44	*p* < 0.01
		Min	−188.69 ± 61.95*	−682.01 ± 126.93	−27.57	*p* < 0.01
		Average	41.70 ± 15.44*	−13.36 ± 62.74	3.81	*p* < 0.01
Iliopsoas	Hip (rad/s^2^)	Max	182.24 ± 48.79*	501.51 ± 109.92	−11.87	*p* < 0.01
		Min	17.99 ± 2.36*	15.56 ± 1.76	3.69	*p* < 0.01
		Average	51.46 ± 9.63*	195.52 ± 47.74	−13.23	*p* < 0.01
	Knee (rad/s^2^)	Max	−5.01 ± 1.46*	−8.70 ± 1.05	9.18	*p* < 0.01
		Min	−127.98 ± 35.74*	−304.45 ± 66.69	10.43	*p* < 0.01
		Average	−44.29 ± 7.93*	−111.52 ± 32.94	8.87	*p* < 0.01
	Ankle (rad/s^2^)	Max	105.35 ± 26.92*	218.09 ± 92.47	−5.24	*p* < 0.01
		Min	−81.19 ± 30.48	−74.03 ± 21.44	−0.86	*p* = 0.40
		Average	−0.51 ± 2.98*	40.72 ± 10.68	−16.63	*p* < 0.01
Quadriceps	Hip (rad/s^2^)	Max	40.09 ± 9.99*	108.03 ± 71.09	−4.23	*p* < 0.01
		Min	−151.35 ± 8.55*	−241.27 ± 64.41	6.19	*p* < 0.01
		Average	−51.20 ± 6.96*	−69.70 ± 24.00	3.31	*p* < 0.01
	Knee (rad/s^2^)	Max	359.24 ± 40.04*	501.54 ± 102.05	−5.81	*p* < 0.01
		Min	65.17 ± 12.24	61.65 ± 9.14	1.03	*p* = 0.31
		Average	146.69 ± 11.26*	222.13 ± 42.60	−7.66	*p* < 0.01
	Ankle (rad/s^2^)	Max	470.30 ± 71.29	504.69 ± 107.93	−1.19	*p* = 0.24
		Min	−289.78 ± 16.52*	−910.23 ± 163.43	16.65	*p* < 0.01
		Average	41.62 ± 11.32*	−120.35 ± 45.68	15.39	*p* < 0.01
Hamstring	Hip (rad/s^2^)	Max	−47.67 ± 18.89	−12.21 ± 59.54	−2.54	*p* = 0.02
		Min	−190.95 ± 29.77*	−521.19 ± 87.17	16.03	*p* < 0.01
		Average	−105.61 ± 17.43*	−168.17 ± 61.28	4.39	*p* < 0.01
	Knee (rad/s^2^)	Max	21.53 ± 7.85*	73.98 ± 23.67	−9.41	*p* < 0.01
		Min	−125.45 ± 19.97	−174.58 ± 66.48	3.17	*p* < 0.01
		Average	−44.46 ± 6.14*	−37.97 ± 7.98	−2.88	*p* < 0.01
	Ankle (rad/s^2^)	Max	62.08 ± 23.64*	174.74 ± 74.80	−6.42	*p* < 0.01
		Min	−84.40 ± 19.46*	−120.22 ± 42.71	3.41	*p* < 0.01
		Average	−17.83 ± 6.80*	−9.66 ± 10.36	−2.95	*p* < 0.01
Soleus	Hip (rad/s^2^)	Max	180.05 ± 81.84*	110.84 ± 37.21	3.44	*p* < 0.01
		Min	−144.92 ± 13.96*	−220.98 ± 30.89	13.99	*p* < 0.01
		Average	−24.52 ± 9.72*	−51.53 ± 11.98	7.83	*p* < 0.01
	Knee (rad/s^2^)	Max	250.84 ± 28.32	229.39 ± 66.45	1.33	0.19
		Min	−392.79 ± 113.20*	−244.91 ± 95.57	−4.46	*p* < 0.01
		Average	20.96 ± 21.96	34.38 ± 32.55	−1.53	*p* = 0.13
	Ankle (rad/s^2^)	Max	−40.48 ± 23.74*	−13.44 ± 6.97	−4.89	*p* < 0.01
		Min	−1566.10 ± 579.62	−2312.07 ± 1308.97	2.33	*p* = 0.03
		Average	−291.89 ± 66.02*	−495.31 ± 117.72	6.74	*p* < 0.01
Gastrocnemius	Hip (rad/s^2^)	Max	228.98 ± 21.04*	163.53 ± 39.09	6.59	*p* < 0.01
		Min	−29.66 ± 6.82*	−90.44 ± 34.56	7.72	*p* < 0.01
		Average	102.20 ± 20.74*	54.23 ± 22.14	7.07	*p* < 0.01
	Knee (rad/s^2^)	Max	49.86 ± 9.84*	118.14 ± 41.91	−7.09	*p* < 0.01
		Min	−444.01 ± 36.06*	−290.35 ± 95.57	−6.73	*p* < 0.01
		Average	−207.12 ± 33.69*	−91.08 ± 22.11	−12.88	*p* < 0.01
	Ankle (rad/s^2^)	Max	3.00 ± 15.97*	−20.63 ± 9.81	5.64	*p* < 0.01
		Min	−893.44 ± 209.94*	−1555.02 ± 437.91	6.09	*p* < 0.01
		Average	−154.69 ± 39.22*	−359.82 ± 81.64	10.13	*p* < 0.01
Tibialis anterior	Hip (rad/s^2^)	Max	149.19 ± 23.80*	451.99 ± 47.80	−25.36	*p* < 0.01
		Min	−110.75 ± 46.58	−74.59 ± 45.16	−2.49	*p* = 0.02
		Average	2.06 ± 7.21*	93.30 ± 15.38	−24.02	*p* < 0.01
	Knee (rad/s^2^)	Max	289.75 ± 82.40*	191.62 ± 85.90	3.69	*p* < 0.01
		Min	−251.48 ± 26.95*	−568.68 ± 78.41	17.11	*p* < 0.01
		Average	13.85 ± 6.10*	−98.25 ± 21.03	22.89	*p* < 0.01
	Ankle (rad/s^2^)	Max	2130.89 ± 333.44*	3750.44 ± 796.97	−8.38	*p* < 0.01
		Min	10.87 ± 6.38*	76.20 ± 39.37	−7.33	*p* < 0.01
		Average	324.26 ± 78.91*	906.18 ± 126.66	−17.44	*p* < 0.01

CON, Control group, i.e., non-pregnant women. *, Significant differences in this index between non-pregnant and pregnant women.

Gastrocnemius muscle and tibialis anterior muscle mainly accelerated knee joint extension in pregnant women, while gastrocnemius muscle and tibialis anterior muscle mainly accelerated knee flexion in the control group. In both the pregnancy group and the control group, gastrocnemius and soleus muscle were the main ankle plantarflexion group, tibialis anterior muscle was the main ankle dorsiflexion group, while quadriceps femoris showed the opposite effect. In the pregnancy group, the quadriceps femoris mainly accelerated ankle dorsiflexion, while in the control group, it primarily accelerated ankle plantarflexion. In addition, almost all the induced acceleration of pregnant women were significantly lower than the control group (*p* < 0.01).

## 4 Discussion

This study compared the differences in gait parameters between pregnant and non-pregnant women and revealed the muscle action of their lower limb joints in the sagittal plane during walking by IAA. As expected, walking speed, stride length, stride frequency, thigh acceleration, and leg falling strength were all lower in the pregnant women than in the control group. Pregnant women also showed smaller joint ROM and peak joint moments than we expected. The IAA obtained during the walking stance phase for both groups of participants showed that muscle function remained generally similar, but the contribution to the joints was slightly different, which was also in line with our expectations for the IAA results.

### 4.1 Gait parameters from the portable gait analyzer

Normally, gait parameters are almost unchanged when walking at the most comfortable speed without any interference (Błaszczyk et al., 2011). However, during pregnancy, gait parameters change temporarily with changes in the body (Foti et al., 2000; Branco et al., 2013; Gilleard, 2013; Yoo et al., 2015), which may increase the risk of falls. In a previous study (Błaszczyk et al. (2016), find that 0.14 m/s decrease in walking speed and 0.09 m decrease in stride length, and single support time decreased significantly throughout pregnancy, which is similar to this study, and the results of these studies are consistent with our original research hypothesis, as during pregnancy, the uterus of the pregnant individual enlarges, the center of gravity of the abdomen shifts forward, the pelvis tilts forward, and so on, all of which lead to changes in the woman’s body center of gravity, thereby affecting her gait. Other studies also shown that pregnancy tends to walk slower (Nagy and King, 1983; Branco et al., 2014; Yoo et al., 2015). This change in pace may lead to changes in other gait characteristics (Błaszczyk et al., 2011; Błaszczyk et al., 2016), including shorter strides and longer double support time.

Although there was no statistically significant difference, this study did observe that the swing work and single support time/double support time of women in pregnancy were also slightly lower than those in the control group. According to previously published studies (Foti et al., 2000; Branco et al., 2013; Błaszczyk et al., 2016), pregnancy women showed a 6% decrease in single support time a 20% increased double support time. And In this study, women also show the same trend, which may help to increase the stability (Błaszczyk et al., 2011; Branco et al., 2014) of the gait and reduce the force (Wearing et al., 2006; Błaszczyk et al., 2011) generated during the gait cycle. In this study, pregnant women showed lower thigh acceleration and leg falling strength. This less strenuous gait pattern in pregnant women is considered to maintain a certain degree of stability (Błaszczyk et al., 2011; Ribeiro et al., 2013; Błaszczyk et al., 2016), and help to reduce the energy cost when walking (Nagy and King, 1983; Błaszczyk et al., 2011). Therefore, it is reasonable to speculate that pregnant women are more likely to walk at a slower speed and spend more time in the double stance phase to enhance walking stability. In summary, during pregnancy, changes occur in the body shape, center of gravity, and skeletal structure of the pregnant women. These changes can affect a woman’s gait and balance, and changes in gait parameters have indeed been observed in this study. Therefore, we recommend that pregnant women choose comfortable and stable shoes to ensure adequate support for their feet, and also engage in appropriate exercises to help improve balance and coordination.

### 4.2 Joint angle and joint moment

Fetal growth will result in a change in the center of gravity, which may lead to changes in the kinematics and kinetics of the lower limb gait, resulting in discomfort and pain (Branco et al., 2013). Similar to the results of Foti et al. (2000), in this study, although gait parameters changed significantly in different degrees, the trend of the joint angle of pregnant women during the gait stance phase was still similar to that of the control group, and there was no significant difference in the range of motion of knee joint and ankle joint to the control group. This study observed that the range of motion of the hip joint decreased significantly in the pregnant women, and the degree of hip flexion was higher than that in the control group during gait support. This may be due to the increase in the size of the fetus during pregnancy, which increases the abdominal volume of the pregnant woman and affects the range of motion of the hip joint of the pregnant woman.

In addition, the study also observed an increase in knee flexion and a decrease in knee extension during gait support in the pregnant women. However, this study also found that pregnant women had larger ankle dorsiflexion and minor plantarflexion during gait support. But according to Branco et al. (2013) and Foti et al. (2000), both ankle plantarflexion and dorsiflexion angles were smaller. This difference may be that there are significant differences in physiological changes among pregnant women, even the participants’ gestation periods are similar, it is difficult to standardize (Huang et al., 2002). In short, the work done on the center of gravity during walking may account for nearly half of the metabolic cost (Doke et al., 2005), so this kinematic change of pregnant women may be a strategy to improve exercise efficiency during pregnancy. During pregnancy, the special gait of a pregnant woman may reduce the cost of energy metabolism while walking (Nagy and King, 1983; Błaszczyk et al., 2011; Błaszczyk et al., 2016).

Most studies on the kinetics parameters of walking in pregnant women focus on plantar pressure (Conder et al., 2019; Forczek et al., 2019), and few studies evaluate the lower limb joint moment of pregnant women during walking (Lymbery and Gillerad, 2005; Hagan and Wong, 2010; Forczek and Staszkiewicz, 2012; Branco et al., 2014). In this study, the peak extension and flexion moment of hip and knee joints decreased significantly during walking in pregnant women. The dorsiflexion moment of the ankle joint was significantly lower than that of the control group. Similar results have been observed in the study of Foti et al. (2000) and Huang et al. (2002). Their study showed that ankle plantarflexion moment decreased in pregnant women. In short, it is worth noting that although in this study, the peak moment of other joints in the pregnant women was significantly smaller than that in the control group, but there was no significant difference in the plantarflexion moment of the ankle joint between the control group and the control group. It may be reasonable to speculate that pregnant women have changed their exercise patterns during pregnancy, and pregnant women may have improved the utilization of the ankle joint compared to the hip joint and knee joint. The study by Bagwell et al. also observed that the ankle joint accounted for a greater proportion of the total work in the walking process of pregnant women (Bagwell et al., 2020).

However, this kinematic and kinetics change may also be that pregnant women do not adapt to morphological changes and fear falls (Dunning et al., 2003; Branco et al., 2013). The discussion of this topic is beyond the scope of this study, and more psychological factors can be taken into account in future studies on the gait of pregnant women.

### 4.3 Induced acceleration

To our knowledge, this is the first time to analyze the induced acceleration of walking in pregnant women. In this study, although the induced acceleration of the main muscle groups of the lower extremities during the pregnancy was lower than the control group, the function was generally similar. In a recent study, Bagwell et al. found that pregnant women showed lower peak gluteus maximus muscle activation during walking (Bagwell et al., 2020). Similar to this study, during walking, the muscle strength of the gluteus muscle and the induced acceleration of hip extension was significantly lower than the control group. Studies by Van et al. have shown that the gluteus maximus can provide closure force for sacroiliac joints (van Wingerden et al., 2004). Therefore, Jennifer et al. believe that the decrease in gluteal muscle activation may lead to low back pain or sacroiliac joint disease when pregnant women gain weight and decrease the stability of the pelvis (Feeney et al., 2018; Bagwell et al., 2020). In addition, it is worth noting that this study observed for the first time that the pregnant women relied more on gluteus than quadriceps to extend their knee joints during walking compared with the control group. And the gastrocnemius muscle also shows the opposite effect to the traditional anatomical classification. According to the traditional anatomical classification, the gluteus muscle mainly acts on the hip joint, and the gastrocnemius muscle mainly induces flexion of the knee joint. The induced acceleration analysis can reveal muscle movements different from the traditional anatomical classification (Souza et al., 2022). For example, the gastrocnemius muscle is one of the main muscle groups that cause hip flexion, although it does not cross the hip joint.

In this study, pregnant women’s change in induced acceleration during walking may be related to special gait patterns. Pregnant women are more likely to reduce walking speed, stride length, and single support time to maintain stability during walking. In this gait mode, the angle and moment of the hip and knee joint of pregnant women changed significantly, while the range of motion and metatarsal flexion moment of the ankle joint had no significant difference compared with the control group. It may be reasonable to speculate that pregnant women are more likely to increase the use of ankle plantarflexion to maintain a normal gait. Compared with the traditional anatomy, the functional changes of the gastrocnemius muscle observed in the knee joint may be similar to those found by Souza et al. (2022). Therefore, it is speculated that in the upright state, the antagonism of the soleus muscle and gastrocnemius muscle may have the function of postural stability in the ankle joint (Souza et al., 2022). In this study, the function of the soleus muscle in the pregnant women to induce knee joint extension may also help maintain knee joint stability during walking.

In short, the induced acceleration may be closely related to gait parameters, joint kinematics, and kinetics. An in-depth analysis of induced acceleration may help us better understand the gait rules of pregnant women during pregnancy.

### 4.4 Limitations

There are still some limitations. Firstly, only participants with a gestational age of 23–36 weeks were recruited, so the results did not apply to all pregnant women. In addition, only the sagittal plane was analyzed. In addition, due to space and data volume limitations, this study only assessed the gait support period of the participants. In addition, the growth status of the fetus and the height of the uterus can also affect the kinematic and kinetic data. However, the main purpose of this study is still to investigate the differences between pregnant and non-pregnant individuals. Further analysis may make it difficult to interpret our research results. Therefore, we will consider recruiting more participants of different gestational ages and more in-depth study and analysis of the sagittal, coronal, and cross-sectional planes in future research. The analysis of gait in women during pregnancy is something we have been working on and in future studies we will also consider a review of the complete gait cycle.

## 5 Conclusion

Overall, the study showed that the walking speed, stride length, step frequency, thigh acceleration, and leg falling strength of pregnant women in the pregnant women decreased to varying degrees. In addition, there are signs that the joint motion of the lower extremities of pregnant women in the pregnant women decreases in the sagittal plane, and the joint moment decreases. At the same time, this study analyzed the induced acceleration of the pregnant women during walking for the first time. Compared with the control group, the joint-induced acceleration decreased and the function changed during walking in the third trimester of pregnancy. As pregnancy progresses, the uterus gradually enlarges and protrudes forward, causing the pregnant woman’s center of gravity to shift forward and leading to changes in the pelvic bones and spine. To maintain balance, pregnant women may adjust their gait by changing their stride length or frequency, making it more stable. Therefore, the changes in biomechanical parameters observed in this study may be due to the changes in body structure during pregnancy, including the movement of the center of gravity and changes in muscle and bone structure. However, some low back pain during pregnancy may also be related to greater dependence on gluteal. Understanding the gait differences between pregnant and non-pregnant women will provide information for future research, evidence for the design of equipment such as shoes for pregnant women, the prevention of falls, and the understanding of pain or musculoskeletal diseases during pregnancy.

## Data Availability

The original contributions presented in the study are included in the article/Supplementary Materials, further inquiries can be directed to the corresponding authors.
